# Evaluation of a matrix-assisted laser desorption ionization-time of flight mass spectrometry assisted, selective broth method to screen for vancomycin-resistant enterococci in patients at high risk

**DOI:** 10.1371/journal.pone.0179455

**Published:** 2017-06-13

**Authors:** Tsi-Shu Huang, Susan Shin-Jung Lee, Chia-Chien Lee, Chiu-Yen Chen, Fang-Chen Chen, Bao-Chen Chen, Cheng Len Sy, Kuan-Sheng Wu

**Affiliations:** 1 Division of Microbiology, Department of pathology and laboratory medicine, Kaohsiung Veterans General Hospital, Kaohsiung, Taiwan; 2 Division of Infectious Diseases, Department of Internal Medicine, Kaohsiung Veterans General Hospital, Kaohsiung, Taiwan; 3 School of Medicine, National Yang-Ming University, Taipei, Taiwan; 4 Department of Nursing, Kaohsiung Veterans General Hospital, Kaohsiung, Taiwan; 5 Infection Control Unit, Kaohsiung Veterans General Hospital, Kaohsiung, Taiwan; Seconda Universita degli Studi di Napoli, ITALY

## Abstract

**Background:**

Bile esculin azide with vancomycin (BEAV) medium is a sensitive, but slightly less specific method for vancomycin-resistant enterococci (VRE) screening. Matrix-assisted laser desorption/ionization time-of-flight mass spectrometry (MALDI-TOF MS) is a rapid method for identification of clinical pathogens. This study aimed to assess the performance of a novel combination screening test for VRE, using BEAV broth combined with MALDI-TOF MS.

**Materials and methods:**

Clinical specimens were collected from patients at risk of VRE carriage, and tested by the novel combination method, using selective BEAV broth culture method followed by MALDI-TOF MS identification (SBEAVM). The reference method used for comparison was the ChromID VRE agar method.

**Results:**

A total of 135 specimens were collected from 78 patients, and 63 specimens tested positive for VRE positive using the ChromID VRE method (positive rate 46.7%). The sensitivity, specificity, positive predictive value, and negative predictive value of SBEAVM method after an incubation period of 28 hours were 93.7%, 90.3%, 89.4%, and 94.2%, respectively. The SBEAVM method when compared to the ChromID VRE method had a shorter turnaround time (29 vs. 48–72 hours) and lower laboratory cost ($2.11 vs. $3.23 per test).

**Conclusions:**

This study demonstrates that SBEAVM is a rapid, inexpensive, and accurate method for use in VRE screening.

## Introduction

Vancomycin-resistant enterococci (VRE) are emerging, multidrug-resistant organisms (MDROs) that occasionally cause healthcare-associated infections and outbreaks.[1–3] Once infected by VRE, patients have limited options of antibiotic treatment. Therefore, many infection control interventions were developed to prevent cross-transmission of VRE. One of them is to employ an active surveillance culture (ASC) protocol to screen for patients at high risk for VRE at admission to a hospital or a ward regardless of clinical symptoms.[1, 4–6] Pre-emptive contact precautions are implemented while awaiting the results of ASC.[1, 4]

Conventional methods for detection of VRE rely on culture-based methods for identification of the organisms and determination of the antimicrobial susceptibility. The bile esculin azide with vancomycin (BEAV) medium is a well-established method and serves as the reference method for newly developed techniques.[7–11] The BEAV method is very sensitive but somewhat less specific.[7, 9, 10] Improved methods, such as chromogenic agars or polymerase chain reaction (PCR), were developed to shorten the identification time. Chromogenic agars enabled rapid identification of Enterococci through the characteristic coloring of colonies. Although the performance of these methods is generally satisfactory,[7, 9, 10, 12] further subculture is required for susceptibility testing. PCR-based methods can detect VRE directly from specimens and significantly reduce identification turnaround times.[11, 13–16] However, the higher cost of PCR-based methods is a major obstacle to widespread use.[17, 18] Another disadvantage of this method is the high false positive rate for detection of the *vanB* gene.[8, 11, 14]

The current method used for VRE screening in most laboratories remains culture dependent, with a turnaround time of 48–72 hours. This places patients in pre-emptive contact isolation for at least 2–3 days. Contact isolation has several undesirable drawbacks, including a decreased rate of healthcare worker–patient interactions, adverse psychological impacts, and higher cost.[19, 20] It may also raise ethical concerns when the final culture results are negative.[17] Thus, a laboratory method for VRE screening with a higher accuracy, lower cost, and shorter turnaround time is needed.

Matrix-assisted laser desorption/ionization time-of-flight mass spectrometry (MALDI-TOF MS) is a rapid and inexpensive method which has been widely used in recent years to identify clinical microorganisms.[21–24] The method was reported to detect Enterococci within 30–60 minutes with high accuracy.[22, 25] The application of MALDI-TOF MS to identify bacteria isolated from BEAV medium may increase specificity of the VRE screening process and decrease the turnaround time, but this possibility has been rarely investigated in previous studies.[24,26]

In the present study, a selective BEAV broth containing vancomycin and carbapenems was developed for rapid detection of VRE. For bacterial isolates that grows in BEAV broth, MALDI-TOF MS was applied for subsequent identification to reduce the turnaround time. The aim of our study was to evaluate the performance of selective BEAV broth culture plus use of MALDI-TOF MS (SBEAVM) as a VRE screening method in comparison to that of the widely used ChromID VRE method.

## Materials and methods

### Clinical setting

The study was conducted at the Infectious Disease (ID) Ward of Kaohsiung Veterans General Hospital, a 1408-bed tertiary care hospital in southern Taiwan. The ID Ward has 42 acute care beds. The 3 most common diseases observed in the ward during the study period included urinary tract infection, pneumonia, and dengue fever.

Active surveillance for VRE was routinely performed in the ID ward in selected patients who fulfilled at least one of the following criteria: residence in a long-term care facility; transfer from an acute care hospital after hospitalization for 2–3 weeks; transfer from an intensive care unit; an endotracheal tube or a Foley catheter in place; open draining wounds; or previous VRE colonization or infection in the past 6 months. Stool specimens were collected for VRE screening. Additional specimens collected included the urine, wound, and pleural effusion from patients with Foley tubes, open draining wounds, and chest drainage tubes, respectively.

These selected patients were placed in pre-emptive contact isolation while awaiting the results of ASC. Patients testing positive for VRE were transferred to a cohort care area. Follow-up cultures (FUCs) were performed weekly until three consecutive cultures tested negative. The study protocol was approved by the Institutional Review Board of Kaohsiung Veterans General Hospital. (No. 16-CT12-19) The informed consent was waived because this study focused on laboratory methodology, and did not collect or record any personal data.

### Specimen collection and processing

**Fig 1:**
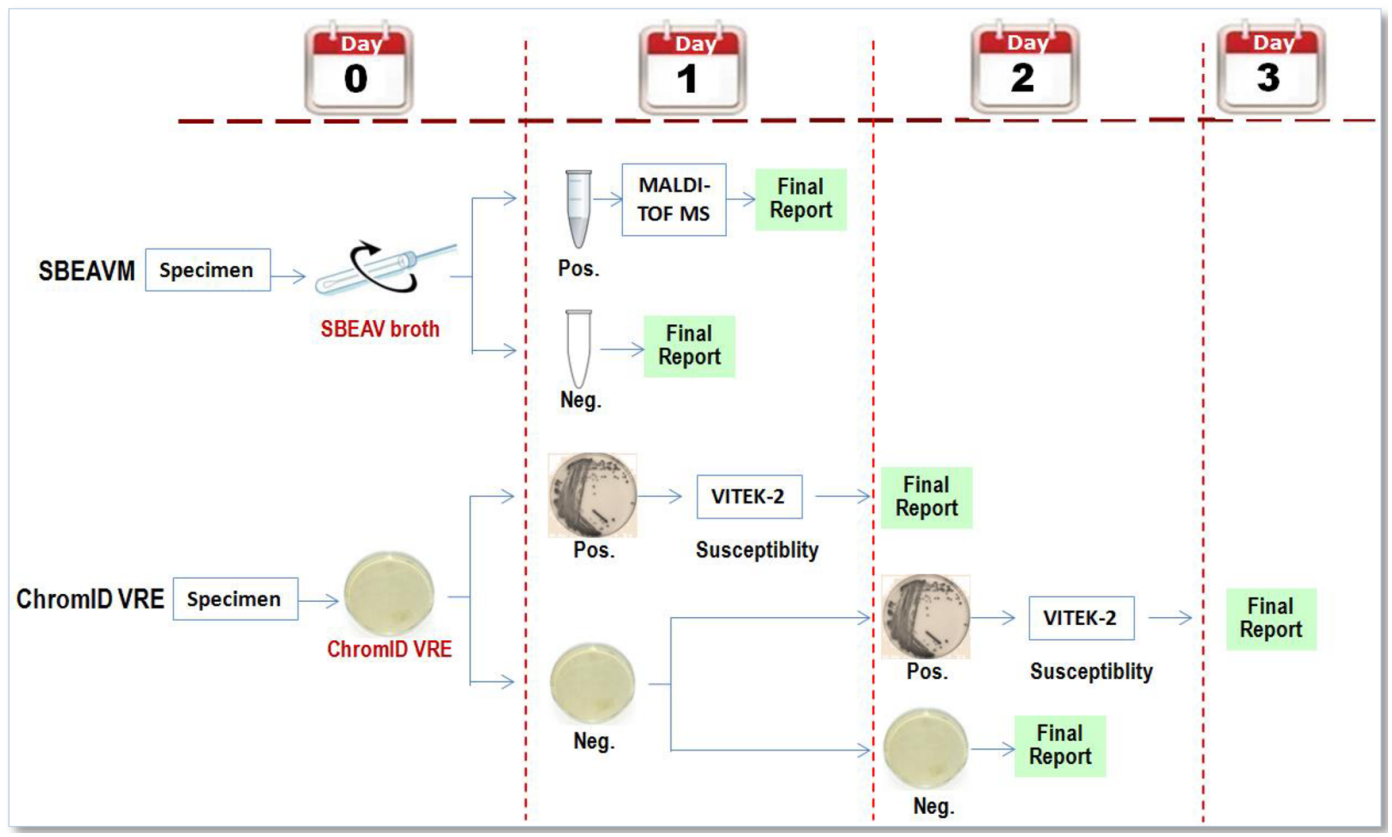
Flow diagram of the two methods used for vancomycin-resistant enterococci (VRE) screening in this study.

The specimens were collected from patients who received routine ASCs and FUCs at the ID ward from November 2015 through February 2016. Two samples were collected on Copan Amies sterile transport swabs (Copan Diagnostics, Corona, CA) and transported to the Microbiology Laboratory for the detection of VRE: one was tested on ChromID VRE agar and the other, by the SBEAVM method. The flow diagram of the two methods were illustrated in Fig 1.

### ChromID VRE method

Specimens were plated directly onto ChromID VRE agar (bioMérieux, Marcy l'Etoile, France). After 24–48 hours of incubation, any purple (*Enterococcus faecium*) or blue (*Enterococcus faecalis*) colonies were subcultured onto a non-selective medium for antimicrobial susceptibility testing using the Vitek 2 AST P602 card in the Vitek 2 system (bioMérieux, Hazelwood, MO). Negative results were not reported until 48 hours. Microorganisms used as controls were *E*. *faecalis* ATCC29212, QC-06A *E*. *faecalis* ATCC51299, and vancomycin-resistant *E*. *faecium*. According to the package insert, the sensitivity and specificity of ChromID VRE agar at day 2 without enrichment is 97.4% and 95.6%, respectively.

### Selective BEAV broth plus MALDI-TOF MS identification (SBEAVM) method

#### SBEAV

A swab was put into 0.5 mL of sterile NaCl 0.9% and thoroughly vortexed, and 50 μl of this solution was inoculated into 0.5 ml of a selective BEAV broth. The BEAV selective broth was prepared by adding vancomycin 16 μg/ml (Chem-Impex International, Inc., Wood Dale, IL),[27] imipenem 8 μg/ml (Mast Diagnostics Group Ltd, Merseyside, UK), and meropenem 8 μg/ml (Sumitomo Pharmaceuticals Co., Ltd., Osaka, Japan) to Bile Esculin Azide broth (Enterococcosel broth, BD, Sparks, MD). The broth was observed for bacterial growth after an incubation period of 20, 24, 28, 32, and 48 hours. The development of gray to black color in the broth indicated a positive result.

#### Sample preparation for MALDI-TOF

Positive broths were centrifuged at 1000 rpm for 1 minute. The supernatant was discarded, and the pellet was resuspended in 0.5 ml of 70% alcohol, vortexed, washed once in 0.5 ml of distilled water, and centrifuged at 14000 rpm for 1 minute. The pellet was spotted onto a MALDI-TOF target plate (direct method). If the direct method failed to show a single identification, further extraction will be done before repeating MALDI-TOF. The remaining pellet will be mixed with 5 μL of acetonitrile and 5 μL of 20% trifluoroacetic acid, and incubated for 15 minutes at room temperature, followed by centrifugation at 14000 rpm for 1 minute. After that, 2 μl of supernatant was spotted onto a MALDI-TOF target plate (extraction method). Each deposit on the target plate was overlaid with 2 μl of matrix solution (saturated solution of α-HCCA [alpha-cyano-4-hydroxycinnamic acid] in 50% acetonitrile and 2.5% trifluoroacetic acid). The matrix-sample was crystallized by air drying at room temperature for 5 minutes.

#### MALDI-TOF MS

Following sample preparation, samples were analyzed with the Vitek MS MALDI-TOF mass spectrometer (bioMérieux, Marcy l'Etoile, France) equipped with a nitrogen laser (l = 337 nm) in linear positive-ion mode, across the mass-to-charge ratio range of 2,000 to 20,000 Da. Each spot was irradiated with 500 laser shots at 50 Hz. Target plates were calibrated and quality controlled both before and after data acquisition by using *Escherichia coli* ATCC 8739.

After the acquisition of spectra, data were transferred from the Vitek MS acquisition station to the Vitek MS analysis server, and identification results were displayed using Myla v2.4 middleware. The total processing and data analysis time was approximately 20 minutes for a single isolate; this time increased by approximately 1 minute for each subsequent sample.

The Vitek MS identification system is based on Vitek MS v2.0 database. This database was built by spectra of known strains. Based on this representative data collection, a weight is assigned to each peak for each species according to its specificity. As part of the identification process, the software compares the spectrum obtained with peak weights defined for each claimed species. The resulting quantitative value, the confidence value, is calculated and expresses the similarity between the unknown organism and every organism or organism group in the database.

A single identification is displayed as a green light when only one significant organism or organism group is retained with a confidence value from 60.0 to 99.9. A “Low-discrimination” identification is displayed as a yellow light when more than one, but not more than four, significant organisms or organism groups are retained. When more than four organisms or organism groups are found, or when no match is found, the organism is considered unidentified, which is indicated as a red light.

## Results

A total of 135 specimens were collected from 78 patients from November 2015 through January 2016. Of the 135 specimens, 95 were stool (70.4%), 30 were urine (22.2%), 7 were wound specimens (5.2%), and the other 3 were pleural fluid (2.2%). The ChromID VRE method identified 63 specimens (46.7%) as positive for VRE.

After incubation for 28 hours, selective BEAV broths detected growth in 72 specimens. MALDI-TOF MS identified 61.1% (44/72) of these specimens as VRE using the direct method. The identification rate for VRE was increased to 97.2% (70/72) using the extraction method for the other 28 specimens initially negative by the direct method.

The performance of the SBEAVM method was optimal after a 28-h incubation, compared to after a 20-h or 24-h incubation. A longer incubation period did not increase the number of positive specimens. The sensitivity, specificity, positive predictive value, and negative predictive value of the 28-h SBEAVM method were 93.7%, 90.3%, 89.4%, and 94.2%, respectively. A detailed assessment of selective BEAV and SBEAVM methods is shown in Table 1. In the end, the seven ChromID-negative specimens identified by the SBEAVM method, and the four false negative specimens detected only by the ChromID VRE method were all *E*. *faecium*.

**Table 1:** Comparison of the selective BEAV broth (SBEAV) and selective BEAV broth plus MALDI-TOF MS identification (SBEAVM) methods at incubation periods of 20, 24, and 28 hours.

Incubation period (hours)	Methods	Number of				Sensitivity (%)	Specificity (%)	PPV (%)	NPV (%)
		TP	FP	TN	FN				
20	SBEAV	32	1	71	31	50.8	98.6	97.0	69.6
	SBEAVM	31	1	71	32	49.2	98.6	96.9	68.9
24	SBEAV	58	9	63	5	92.1	87.5	86.6	92.7
	SBEAVM	56	6	66	7	88.9	91.7	90.3	90.4
28	SBEAV	61	11	61	2	96.8	84.7	84.7	96.8
	SBEAVM	59	7	65	4	93.7	90.3	89.4	94.2

TP findings were defined as the development of gray to black color in BEAV broth (and furthered identified as *E*. *faecium* or *E*. *faecalis* by MALTI-TOF MS), and were confirmed by the ChromID VRE method

FP findings were defined as the development of gray to black color in BEAV broth (and furthered identified as *E*. *faecium* or *E*. *faecalis* by MALTI-TOF MS) with negative ChromID VRE results

TN findings were defined as no typical color change (or typical color change but identified as bacteria other than *E*. *faecium* or *E*. *faecalis* by MALTI-TOF MS) with negative ChromID VRE results

FN findings were defined as no typical color change (or typical color change but identified as bacteria other than *E*. *faecium* or *E*. *faecalis* by MALTI-TOF MS) with positive ChromID VRE results

SBEAV, selective bile esculin azide broth with vancomycin; MALDI-TOF MS, matrix-assisted laser desorption/ionization time-of-flight mass spectrometry; SBEAVM, selective BEAV broth plus MALDI-TOF MS identification; VRE, vancomycin-resistant *Enterococci*; TP, true positive; FP, false positive; TN, true negative; FN, false negative

ASC was performed on 72 (53.3%) specimens and FUC was performed on 63 (46.7%) specimens. The rate of VRE colonization detected by FUCs was higher than that detected by ASCs (68.3% vs. 27.8%). Table 2 shows the results of ASC and FUC using the SBEAVM method to detect VRE.

**Table 2:** Assessment of the selective BEAV broth plus MALDI-TOF MS (SBEAVM) method for the detection of VRE in selected patients.

Incubation period (hours)	Purpose	Number of				Sensitivity (%)	Specificity (%)	PPV (%)	NPV (%)
		TP	FP	TN	FN				
20	ASC	11	0	52	9	55.0	100.0	100.0	85.3
	FUC	20	1	19	23	46.5	95.0	95.2	45.2
24	ASC	16	2	50	4	80	96.2	88.9	92.6
	FUC	40	4	16	3	93.0	80.0	90.9	84.2
28	ASC	18	3	49	2	90.0	94.2	85.7	96.1
	FUC	41	4	16	2	95.3	80.0	91.1	88.9

The screening method was used for the ASC of high-risk patients without VRE colonization or infection during the previous 6 months.

The screening method was used for the FUC of patients with VRE colonization or infection during the previous 6 months.

BEAV, bile esculin azide broth with vancomycin; MALDI-TOF MS, matrix-assisted laser desorption/ionization time-of-flight mass spectrometry; SBEAVM, selective BEAV broth plus MALDI-TOF MS identification; VRE, vancomycin-resistant *Enterococci*; TP, true positive; FP, false positive; TN, true negative; FN, false negative; ASC, active surveillance culture; FUC, follow-up culture

The cost per test of the selective BEAV broth method, MALDI-TOF direct method, and MALDI-TOF extraction method was $0.30, $2.20, and $2.80, respectively. The cost of instruments and technicians staff was not included in the analysis for both methods. As for the ChromID VRE method, the cost of each agar plate and subsequent identification was $0.60 and $5.60, respectively. On average, the cost of using the SBEAVM method was only 65.3% of that using the ChromID VRE method (cost per test: SBEAVM $2.11 vs. ChromID VRE $3.23; Table 3).

**Table 3:** Comparison of laboratory material costs and turnaround times between the selective BEAV broth plus MALDI-TOF MS (SBEAVM) and ChromID VRE methods for VRE detection in 135 specimens.

	SBEAVM	ChromID VRE
ASC (n = 72)		
Total cost (USD)	110.91	155.87
Cost per test (USD)	1.54	2.16
FUC (n = 63)		
Total cost (USD)	173.71	280.03
Cost per test (USD)	2.76	4.44
All specimens (n = 135)		
Total cost (USD)	284.62	435.90
Cost per test (USD)	2.11	3.23
Turnaround time (hours)		
Culture positive	29	72
Culture negative	28	48

BEAV, bile esculin azide broth with vancomycin; MALDI-TOF MS, matrix-assisted laser desorption/ionization time-of-flight mass spectrometry; SBEAVM, selective BEAV broth plus MALDI-TOF MS identification; VRE, vancomycin-resistant *Enterococci*; ASC, active surveillance culture; USD, US dollars; FUC, follow-up culture

The cost of instruments and technicians staff was not included in the analysis for both methods.

## Discussion

VRE screening using the selective BEAV broth method followed by MALDI-TOF MS identification was as accurate, less costly, and faster (i.e., had a shorter turnaround time) than the ChromID VRE method. When implementing VRE screening as one of the infection control strategies, the balance between accuracy, cost, turnaround time, and technique availability is an important, but never easy task. Our results demonstrated the feasibility and advantages of the SBEAVM method as an acceptable substitute to ChromID VRE method, which is commonly used in healthcare facilities. Subgroup analysis further revealed that the SBEAVM method was suitable both for active surveillance and for follow up purposes. The results warrant large-scale application of the method in the future.

Use of BEAV plus MALDI-TOF MS identification method for VRE screening has been reported before.[24, 26] However, our SBEAVM method was different from previous studies in several aspects. First, to increase the specificity of the BEAV method, two carbapenems were added to the broth to better inhibit growth of gram negative bacteria, in addition to the bile in BEAV which served as an inhibitor. Second, our comparator was ChromID VRE method, not conventional phenotypic identification,[24] Vitek 2 or API 20 STREP.[26] Third, the database we used for identification was Vitek v2.0, which is approved by the US Federal and Drug Administration for clinical use, while the database used in the previous study was for research use only.[24] In conclusion, our study proposed a better solution to VRE screening in the real world.

There are at least two advantages of the SBEAVM method over the ChromID VRE method. First, the turnaround time for the SBEAVM method is shorter, therefore, reducing the time that patients are placed on preemptive contact precautions, and thereby minimizing the side effects and reducing the costs of isolation. We estimated the cost of contact precaution saved per patient using the SBEAVM method when the culture results were positive and negative was $14.9 and $6.6, respectively. Second, use of the SBEAVM method lowers laboratory cost, which makes implementation of the ASC policy more affordable. In addition, we recommend the universal adoption of the extraction method to prepare specimens for MALDI-TOF analysis to further decrease cost and turnaround time. Specimen preparation using the extraction method is more cost effective and time saving. The reason is that although direct method costs a little less than the extraction method, extraction is needed in more than one third (36.1%) of positive specimens before identification is possible.

In subgroup analysis, the SBEAVM method was more specific when used for ASC, and more sensitive when used for FUC. The results are welcome for both purposes. We believe that specificity is more important than sensitivity when the method is used for active surveillance. A higher specificity means fewer false positives and lower rate of unnecessary contact precautions and use of cohorting care. In particular, isolating patients without VRE carriage in VRE cohorting areas raises the issue of patient safety, which may hamper future implementation of the policy. On the contrary, a high sensitivity is more crucial when the method is used for FUC. A higher sensitivity implies a lower false negative rate and avoids the removal of contact precautions from patients who still carry VRE. Overall, our results support use of the SBEAVM method for both ASC and FUC purposes.

In the study, there were two and four false negative specimens by SBEAV and SBEAVM method, respectively. We have two possible explanations. First, false negative results of the SBEAVM method may be due to false positive specimens of ChromID VRE method. There were two specimens showed typical color changes on both ChromID VRE and SBEAV broth, and were identified as bacteria other than *E*. *faecium* or *E*. *faecalis* by MALTI-TOF MS. These two specimens may be falsely identified by the ChromID VRE method. Second, although two samples were collected at the same time, the bacteria density of two swabs may not be the same. Less bacteria amount may decrease the sensitivity of the laboratory method.

The study has two limitations. First, the study did not use a third method with higher accuracy, such as PCR-based or sequencing techniques, as the reference method. Those specimens considered false positive, that is, specimens positive by the SBEAVM method but negative by the ChromID VRE method, may be true VRE. That is, the specificity of the SBEAVM method may be underestimated. On the other hand, it also made further analysis of the reasons for false negative specimens difficult. Second, the majority of specimens in the study were stool and urine. The utility of SBEAVM method to detect VRE in other specimen types, such as sputum, blood, or cerebrospinal fluid still needs further evaluation.

To conclude, the accuracy of the SBEAVM method is comparable to that of the ChromID method, which is widely used for VRE screening. The advantages of low cost and short turnaround time make the SBEAVM method more suitable for VRE detection.

## Supporting information

S1 Dataset The raw dataset showing the results of the two laboratory methods in this study. (XLSX)
